# Low-temperature redetermination of 4-chloro-2-[tris­(hydroxy­meth­yl)methyl­imino­meth­yl]phenol as zwitterionic 4-chloro-2-[tris­(hydroxy­meth­yl)methyl­iminiometh­yl]phenolate

**DOI:** 10.1107/S160053680803866X

**Published:** 2008-11-26

**Authors:** Seik Weng Ng

**Affiliations:** a Department of Chemistry, University of Malaya, 50603 Kuala Lumpur, Malaysia

## Abstract

The title Schiff base, C_11_H_14_ClNO_4_, originally refined as a neutral mol­ecule [Chumakov, Antosyak, Mazus, Tsapkov & Samus (2000), *Crystallogr. Rep.* **45**, 945–950], is inter­preted as a zwitterionic compound. There are two independent zwitterions in the asymmetric unit. Five of the six hydr­oxy groups of the –CH_2_OH fragments are disordered. In one zwitterion, two are disordered over three sites [0.53 (1):0.33 (1):0.14 (1) and 0.65 (1):0.18 (1):0.17 (1)] and the third over two sites [0.66 (1):0.34 (1)]. In the second zwitterion, two are disordered over two sites [0.84 (1):0.16 (1) and 0.83 (1):0.17 (1)] and the third hydr­oxy group is ordered.

## Related literature

The room-temperature crystal structure was inter­preted as a neutral mol­ecule; the disordered structure (the disorder confined to the hydr­oxy part of one of the six –CH_2_OH groups) refined to 0.072, see: Chumakov *et al.* (2000 ▶). The unsubstituted parent Schiff base is also a zwitterion; its structure has been determined several times, see: Asgedom *et al.* (1996 ▶); Odabas˛ogˇlu *et al.* (2003 ▶); Tatar *et al.* (2005 ▶); Zhang *et al.* (2000 ▶).
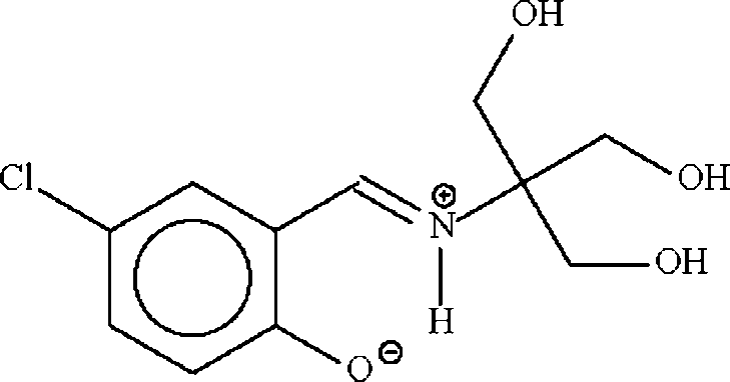


## Experimental

### 

#### Crystal data

C_11_H_14_ClNO_4_
*M* *_r_* = 259.68
Triclinic, 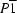

*a* = 7.0174 (1) Å
*b* = 10.1935 (1) Å
*c* = 16.7234 (2) Å
α = 79.520 (1)°
β = 89.031 (1)°
γ = 85.258 (1)°
*V* = 1172.27 (2) Å^3^
*Z* = 4
Mo *K*α radiation
μ = 0.33 mm^−1^
*T* = 100 (2) K
0.30 × 0.20 × 0.05 mm

#### Data collection

Bruker SMART APEX diffractometer
Absorption correction: multi-scan (*SADABS*; Sheldrick, 1996 ▶) *T*_min_ = 0.908, *T*_max_ = 0.984
11241 measured reflections
5361 independent reflections
4771 reflections with *I* > 2σ(*I*)
*R*_int_ = 0.018

#### Refinement

*R*[*F* ^2^ > 2σ(*F* ^2^)] = 0.042
*wR*(*F* ^2^) = 0.117
*S* = 1.01
5361 reflections
360 parameters
47 restraints
H atoms treated by a mixture of independent and constrained refinement
Δρ_max_ = 0.41 e Å^−3^
Δρ_min_ = −0.74 e Å^−3^

### 

Data collection: *APEX2* (Bruker, 2007 ▶); cell refinement: *SAINT* (Bruker, 2007 ▶); data reduction: *SAINT*; program(s) used to solve structure: *SHELXS97* (Sheldrick, 2008 ▶); program(s) used to refine structure: *SHELXL97* (Sheldrick, 2008 ▶); molecular graphics: *X-SEED* (Barbour, 2001 ▶); software used to prepare material for publication: *publCIF* (Westrip, 2008 ▶).

## Supplementary Material

Crystal structure: contains datablocks global, I. DOI: 10.1107/S160053680803866X/tk2330sup1.cif

Structure factors: contains datablocks I. DOI: 10.1107/S160053680803866X/tk2330Isup2.hkl

Additional supplementary materials:  crystallographic information; 3D view; checkCIF report

## Figures and Tables

**Fig. 1.**
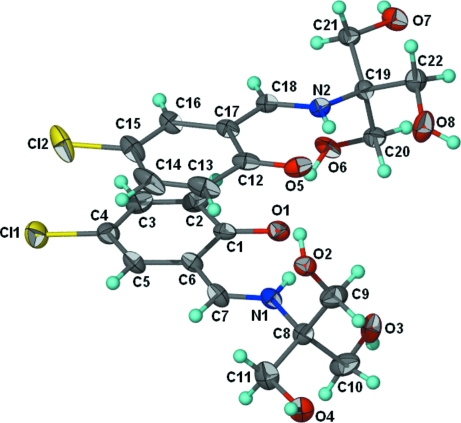
Thermal ellipsoid plot (Barbour, 2001) of C22H28Cl2N2O8 at the 70% probability level. Hydrogen atoms are drawn as spheres of arbitrary radius. The minor disorder components are not shown for clarity.

## supplementary crystallographic information

### Comment

A reevaluation of the title compound, originally reported as a neutral molecule (Chumakov *et al.*, 2000), shows a zwitterionic compound, at low temperature, Fig. 1.

### Experimental

The compound was synthesized from the reaction of 5-chlorosalicylaldehyde and tris(hydroxymethyl)aminomethane (Chumakov *et al.*, 2000), and was recrystallized from ethanol.

### Refinement

Five of the six hydroxy groups of the –CH_2_OH fragments are disordered. In one zwitterion, two are disordered over three sites [0.53 (1): 0.33: 0.14] and the third over two sites [0.65 (1): 0.18: 0.17]. In the second zwitterion, two are disordered over two sites [0.84 (1): 0.16 and 0.83 (1): 0.17] and the third is ordered. For each disordered –CH_2_OH fragment, the C–O distances (of the unprimed and primed atoms) were restrained to within 0.01 Å. The temperature factors of the primed atom(s) were restrained to those of the unprimed ones.

Carbon- and oxygen-bound hydrogen atoms were placed at calculated positions (C–H 0.95–0.99, O–H 0.84 Å) and were treated as riding on their parent atoms, with *U*(H) set to 1.2–1.5 times *U*_eq_(*C*,*O*). The iminium hydrogen atoms were located in a difference Fourier map, and were refined with a distance restraint of N–H 0.88±0.01 Å; their temperature factors were freely refined.

Owing to the disorder, hydrogen bonding interactions involving the hydroxyl groups were not computed.

### Crystal data

**Table:**

C_11_H_14_ClNO_4_	*Z* = 4
*M**_r_* = 259.68	*F*_000_ = 544
Triclinic, *P*1	*D*_x_ = 1.471 Mg m^−^^3^
Hall symbol: -P 1	Mo *K*α radiation λ = 0.71073 Å
*a* = 7.0174 (1) Å	Cell parameters from 6864 reflections
*b* = 10.1935 (1) Å	θ = 2.2–28.3º
*c* = 16.7234 (2) Å	µ = 0.33 mm^−^^1^
α = 79.520 (1)º	*T* = 100 (2) K
β = 89.031 (1)º	Prism, yellow
γ = 85.258 (1)º	0.30 × 0.20 × 0.05 mm
*V* = 1172.27 (2) Å^3^	

### Data collection

**Table:**

Bruker SMART APEX diffractometer	5361 independent reflections
Radiation source: fine-focus sealed tube	4771 reflections with *I* > 2σ(*I*)
Monochromator: graphite	*R*_int_ = 0.018
*T* = 100(2) K	θ_max_ = 27.5º
ω scans	θ_min_ = 1.2º
Absorption correction: Multi-scan(SADABS; Sheldrick, 1996)	*h* = −9→9
*T*_min_ = 0.908, *T*_max_ = 0.984	*k* = −13→13
11241 measured reflections	*l* = −21→21

### Refinement

**Table:**

Refinement on *F*^2^	Secondary atom site location: difference Fourier map
Least-squares matrix: full	Hydrogen site location: inferred from neighbouring sites
*R*[*F*^2^ > 2σ(*F*^2^)] = 0.042	H atoms treated by a mixture of independent and constrained refinement
*wR*(*F*^2^) = 0.117	*w* = 1/[σ^2^(*F*_o_^2^) + (0.0573*P*)^2^ + 1.0948*P*] where *P* = (*F*_o_^2^ + 2*F*_c_^2^)/3
*S* = 1.01	(Δ/σ)_max_ = 0.001
5361 reflections	Δρ_max_ = 0.41 e Å^−^^3^
360 parameters	Δρ_min_ = −0.74 e Å^−^^3^
47 restraints	Extinction correction: none
Primary atom site location: structure-invariant direct methods	

### Fractional atomic coordinates and isotropic or equivalent isotropic displacement parameters (Å^2^)

**Table:**

	*x*	*y*	*z*	*U*_iso_*/*U*_eq_	Occ. (<1)
Cl1	0.74259 (7)	0.67565 (5)	0.56473 (3)	0.03176 (13)	
Cl2	0.23984 (7)	0.77985 (6)	0.56151 (3)	0.03809 (15)	
O1	0.54240 (19)	0.73990 (13)	0.21969 (8)	0.0239 (3)	
O5	0.03446 (18)	0.95420 (12)	0.21807 (8)	0.0246 (3)	
O2	0.1961 (3)	1.1785 (2)	0.17629 (14)	0.0204 (4)	0.535 (2)
H2	0.1471	1.1063	0.1939	0.031*	0.535 (2)
O2'	0.2372 (5)	1.0814 (4)	0.0932 (2)	0.0204 (4)	0.328 (3)
H2'	0.1688	1.0397	0.1292	0.031*	0.328 (3)
O2"	0.3318 (13)	1.2743 (9)	0.0813 (5)	0.0204 (4)	0.137 (3)
H2"	0.4077	1.3189	0.1015	0.031*	0.137 (3)
O3	0.6388 (3)	0.98149 (19)	0.05581 (12)	0.0256 (4)	0.650 (3)
H3	0.7445	0.9411	0.0476	0.038*	0.650 (3)
O3'	0.8488 (9)	1.1295 (10)	0.0883 (6)	0.0256 (4)	0.183 (6)
H3'	0.8968	1.0856	0.1317	0.038*	0.183 (6)
O3"	0.8481 (9)	1.0984 (11)	0.0690 (7)	0.0256 (4)	0.166 (6)
H3"	0.8995	1.0227	0.0894	0.038*	0.166 (6)
O4	0.6047 (3)	1.35087 (18)	0.13329 (12)	0.0254 (5)	0.660 (5)
H4	0.5375	1.4132	0.1492	0.038*	0.660 (5)
O4'	0.5213 (6)	1.2777 (4)	0.2551 (2)	0.0254 (5)	0.34
H4'	0.4631	1.3531	0.2394	0.038*	0.340 (5)
O6	0.3862 (2)	0.54296 (15)	0.17552 (10)	0.0241 (5)	0.835 (6)
H6	0.4403	0.6031	0.1925	0.036*	0.835 (6)
O6'	0.2819 (11)	0.5226 (7)	0.0539 (5)	0.0241 (5)	0.16
H6'	0.1780	0.4913	0.0462	0.036*	0.165 (6)
O7	−0.0249 (2)	0.40172 (15)	0.12281 (10)	0.0251 (5)	0.831 (6)
H7	0.0171	0.3226	0.1412	0.038*	0.831 (6)
O7'	0.1139 (11)	0.3978 (7)	0.2403 (4)	0.0251 (5)	0.17
H7'	0.2264	0.4154	0.2276	0.038*	0.169 (6)
O8	−0.0449 (2)	0.81865 (13)	0.05073 (8)	0.0336 (3)	
H8	−0.0069	0.8306	0.0023	0.050*	
N1	0.5472 (2)	0.99524 (14)	0.21010 (9)	0.0194 (3)	
H1N	0.537 (4)	0.9209 (17)	0.1907 (15)	0.043 (7)*	
N2	0.0469 (2)	0.70644 (14)	0.20403 (9)	0.0174 (3)	
H2N	0.029 (3)	0.7936 (11)	0.1854 (14)	0.033 (6)*	
C1	0.5840 (2)	0.72652 (17)	0.29646 (11)	0.0192 (3)	
C2	0.6121 (3)	0.59874 (18)	0.34689 (12)	0.0271 (4)	
H2A	0.5990	0.5209	0.3243	0.032*	
C3	0.6579 (3)	0.58514 (18)	0.42746 (12)	0.0268 (4)	
H3A	0.6760	0.4983	0.4598	0.032*	
C4	0.6782 (2)	0.69780 (18)	0.46259 (11)	0.0232 (4)	
C5	0.6502 (2)	0.82333 (18)	0.41675 (11)	0.0224 (3)	
H5	0.6619	0.8996	0.4411	0.027*	
C6	0.6041 (2)	0.84008 (16)	0.33387 (11)	0.0188 (3)	
C7	0.5840 (2)	0.97254 (17)	0.28681 (11)	0.0216 (3)	
H7A	0.5984	1.0463	0.3131	0.026*	
C8	0.5387 (2)	1.12384 (17)	0.15371 (10)	0.0203 (3)	
C9	0.3328 (3)	1.1578 (2)	0.12391 (13)	0.0329 (4)	
H9A	0.3316	1.2392	0.0813	0.039*	0.535 (2)
H9B	0.2978	1.0842	0.0972	0.039*	0.535 (2)
H9C	0.2577	1.1804	0.1709	0.039*	0.328 (3)
H9D	0.3335	1.2417	0.0834	0.039*	0.328 (3)
H9E	0.2957	1.0929	0.0908	0.039*	0.137 (3)
H9F	0.2433	1.1569	0.1704	0.039*	0.137 (3)
C10	0.6699 (3)	1.1019 (2)	0.08285 (13)	0.0338 (5)	
H10A	0.6471	1.1782	0.0373	0.041*	0.650 (3)
H10B	0.8048	1.0987	0.1000	0.041*	0.650 (3)
H10C	0.6697	1.0070	0.0769	0.041*	0.183 (6)
H10D	0.6146	1.1573	0.0325	0.041*	0.183 (6)
H10E	0.6352	1.0150	0.0712	0.041*	0.166 (6)
H10F	0.6163	1.1698	0.0371	0.041*	0.166 (6)
C11	0.6064 (3)	1.23560 (18)	0.19279 (12)	0.0289 (4)	
H11A	0.5204	1.2514	0.2382	0.035*	0.660 (5)
H11B	0.7372	1.2106	0.2146	0.035*	0.660 (5)
H11C	0.7408	1.2083	0.2092	0.035*	0.340 (5)
H11D	0.6095	1.3150	0.1489	0.035*	0.340 (5)
C12	0.0782 (2)	0.91510 (17)	0.29366 (12)	0.0216 (3)	
C13	0.1062 (3)	1.00731 (19)	0.34634 (14)	0.0292 (4)	
H13	0.0916	1.1006	0.3253	0.035*	
C14	0.1531 (3)	0.9650 (2)	0.42592 (15)	0.0335 (5)	
H14	0.1714	1.0291	0.4593	0.040*	
C15	0.1751 (3)	0.8282 (2)	0.45953 (12)	0.0271 (4)	
C16	0.1489 (2)	0.73448 (18)	0.41255 (11)	0.0219 (3)	
H16	0.1619	0.6419	0.4357	0.026*	
C17	0.1027 (2)	0.77585 (16)	0.32963 (11)	0.0187 (3)	
C18	0.0882 (2)	0.67582 (16)	0.28079 (10)	0.0175 (3)	
H18	0.1095	0.5842	0.3053	0.021*	
C19	0.0507 (2)	0.61584 (16)	0.14497 (10)	0.0172 (3)	
C20	0.2570 (2)	0.60202 (17)	0.11302 (11)	0.0213 (3)	
H20A	0.2621	0.5465	0.0701	0.026*	0.835 (6)
H20B	0.2959	0.6917	0.0884	0.026*	0.835 (6)
H20C	0.2963	0.6924	0.0902	0.026*	0.165 (6)
H20D	0.3425	0.5636	0.1593	0.026*	0.165 (6)
C21	−0.0160 (2)	0.47961 (16)	0.18370 (10)	0.0197 (3)	
H21A	0.0747	0.4344	0.2267	0.024*	0.831 (6)
H21B	−0.1436	0.4914	0.2088	0.024*	0.831 (6)
H21C	−0.1389	0.4944	0.2119	0.024*	0.169 (6)
H21D	−0.0407	0.4299	0.1398	0.024*	0.169 (6)
C22	−0.0847 (3)	0.68282 (17)	0.07586 (11)	0.0229 (3)	
H22A	−0.0676	0.6350	0.0294	0.027*	
H22B	−0.2189	0.6784	0.0947	0.027*	

### Atomic displacement parameters (Å^2^)

**Table:**

	*U*^11^	*U*^22^	*U*^33^	*U*^12^	*U*^13^	*U*^23^
Cl1	0.0346 (3)	0.0301 (2)	0.0279 (2)	−0.00246 (19)	−0.01100 (18)	0.00271 (18)
Cl2	0.0343 (3)	0.0474 (3)	0.0384 (3)	0.0139 (2)	−0.0164 (2)	−0.0295 (2)
O1	0.0282 (7)	0.0225 (6)	0.0243 (6)	−0.0092 (5)	0.0037 (5)	−0.0100 (5)
O5	0.0241 (6)	0.0153 (6)	0.0333 (7)	−0.0013 (5)	0.0054 (5)	−0.0018 (5)
O2	0.0200 (9)	0.0169 (9)	0.0237 (9)	−0.0017 (7)	−0.0014 (7)	−0.0023 (7)
O2'	0.0200 (9)	0.0169 (9)	0.0237 (9)	−0.0017 (7)	−0.0014 (7)	−0.0023 (7)
O2"	0.0200 (9)	0.0169 (9)	0.0237 (9)	−0.0017 (7)	−0.0014 (7)	−0.0023 (7)
O3	0.0374 (10)	0.0208 (9)	0.0184 (9)	0.0056 (7)	0.0022 (7)	−0.0069 (7)
O3'	0.0374 (10)	0.0208 (9)	0.0184 (9)	0.0056 (7)	0.0022 (7)	−0.0069 (7)
O3"	0.0374 (10)	0.0208 (9)	0.0184 (9)	0.0056 (7)	0.0022 (7)	−0.0069 (7)
O4	0.0304 (10)	0.0163 (8)	0.0289 (10)	0.0014 (7)	−0.0012 (7)	−0.0035 (7)
O4'	0.0304 (10)	0.0163 (8)	0.0289 (10)	0.0014 (7)	−0.0012 (7)	−0.0035 (7)
O6	0.0191 (8)	0.0217 (8)	0.0350 (9)	−0.0028 (6)	−0.0028 (6)	−0.0135 (6)
O6'	0.0191 (8)	0.0217 (8)	0.0350 (9)	−0.0028 (6)	−0.0028 (6)	−0.0135 (6)
O7	0.0273 (9)	0.0185 (8)	0.0326 (9)	−0.0019 (6)	0.0009 (6)	−0.0125 (6)
O7'	0.0273 (9)	0.0185 (8)	0.0326 (9)	−0.0019 (6)	0.0009 (6)	−0.0125 (6)
O8	0.0575 (10)	0.0221 (7)	0.0194 (6)	−0.0016 (6)	−0.0017 (6)	0.0000 (5)
N1	0.0198 (7)	0.0167 (7)	0.0231 (7)	−0.0006 (5)	−0.0021 (5)	−0.0075 (5)
N2	0.0191 (7)	0.0132 (6)	0.0200 (7)	−0.0008 (5)	0.0024 (5)	−0.0042 (5)
C1	0.0150 (7)	0.0201 (8)	0.0246 (8)	−0.0050 (6)	0.0038 (6)	−0.0082 (6)
C2	0.0326 (10)	0.0189 (8)	0.0318 (10)	−0.0069 (7)	0.0093 (8)	−0.0088 (7)
C3	0.0286 (9)	0.0187 (8)	0.0315 (10)	−0.0017 (7)	0.0052 (7)	−0.0014 (7)
C4	0.0185 (8)	0.0250 (9)	0.0250 (9)	−0.0013 (6)	−0.0032 (6)	−0.0020 (7)
C5	0.0199 (8)	0.0210 (8)	0.0275 (9)	−0.0013 (6)	−0.0078 (7)	−0.0068 (7)
C6	0.0145 (7)	0.0181 (8)	0.0244 (8)	−0.0002 (6)	−0.0039 (6)	−0.0059 (6)
C7	0.0211 (8)	0.0192 (8)	0.0262 (9)	0.0016 (6)	−0.0069 (6)	−0.0094 (7)
C8	0.0208 (8)	0.0198 (8)	0.0203 (8)	−0.0005 (6)	0.0008 (6)	−0.0044 (6)
C9	0.0230 (9)	0.0364 (11)	0.0336 (10)	−0.0047 (8)	−0.0029 (8)	0.0098 (8)
C10	0.0383 (11)	0.0307 (10)	0.0367 (11)	−0.0115 (8)	0.0183 (9)	−0.0150 (8)
C11	0.0396 (11)	0.0174 (8)	0.0301 (10)	0.0014 (7)	−0.0068 (8)	−0.0066 (7)
C12	0.0132 (7)	0.0175 (8)	0.0360 (10)	−0.0019 (6)	0.0056 (6)	−0.0101 (7)
C13	0.0212 (9)	0.0185 (8)	0.0515 (12)	−0.0012 (7)	−0.0006 (8)	−0.0161 (8)
C14	0.0225 (9)	0.0308 (10)	0.0549 (13)	0.0016 (7)	−0.0065 (8)	−0.0292 (9)
C15	0.0175 (8)	0.0338 (10)	0.0344 (10)	0.0042 (7)	−0.0049 (7)	−0.0202 (8)
C16	0.0174 (8)	0.0230 (8)	0.0273 (9)	0.0019 (6)	−0.0009 (6)	−0.0114 (7)
C17	0.0136 (7)	0.0185 (8)	0.0261 (8)	−0.0013 (6)	0.0024 (6)	−0.0096 (6)
C18	0.0161 (7)	0.0150 (7)	0.0219 (8)	−0.0011 (6)	0.0022 (6)	−0.0052 (6)
C19	0.0191 (8)	0.0158 (7)	0.0176 (7)	−0.0014 (6)	0.0016 (6)	−0.0056 (6)
C20	0.0204 (8)	0.0213 (8)	0.0236 (8)	−0.0034 (6)	0.0042 (6)	−0.0076 (6)
C21	0.0211 (8)	0.0167 (7)	0.0225 (8)	−0.0037 (6)	0.0018 (6)	−0.0054 (6)
C22	0.0251 (9)	0.0214 (8)	0.0215 (8)	0.0007 (7)	−0.0012 (7)	−0.0036 (6)

### Geometric parameters (Å, °)

**Table:**

Cl1—C4	1.7442 (19)	C6—C7	1.431 (2)
Cl2—C15	1.744 (2)	C7—H7A	0.9500
O1—C1	1.302 (2)	C8—C11	1.524 (2)
O5—C12	1.289 (2)	C8—C10	1.526 (2)
O2—C9	1.318 (3)	C8—C9	1.528 (3)
O2—H2	0.8400	C9—H9A	0.9900
O2'—C9	1.251 (4)	C9—H9B	0.9900
O2'—H2'	0.8400	C9—H9C	0.9900
O2"—C9	1.268 (9)	C9—H9D	0.9900
O2"—H2"	0.8400	C9—H9E	0.9900
O3—C10	1.417 (3)	C9—H9F	0.9900
O3—H3	0.8400	C10—H10A	0.9900
O3'—C10	1.318 (6)	C10—H10B	0.9900
O3'—H3'	0.8400	C10—H10C	0.9900
O3"—C10	1.266 (6)	C10—H10D	0.9900
O3"—H3"	0.8400	C10—H10E	0.9900
O4—C11	1.394 (3)	C10—H10F	0.9900
O4—H4	0.8400	C11—H11A	0.9900
O4'—C11	1.313 (4)	C11—H11B	0.9900
O4'—H4'	0.8400	C11—H11C	0.9900
O6—C20	1.411 (2)	C11—H11D	0.9900
O6—H6	0.8400	C12—C13	1.427 (2)
O6'—C20	1.389 (6)	C12—C17	1.435 (2)
O6'—H6'	0.8400	C13—C14	1.360 (3)
O7—C21	1.405 (2)	C13—H13	0.9500
O7—H7	0.8400	C14—C15	1.402 (3)
O7'—C21	1.428 (6)	C14—H14	0.9500
O7'—H7'	0.8400	C15—C16	1.368 (2)
O8—C22	1.421 (2)	C16—C17	1.409 (2)
O8—H8	0.8400	C16—H16	0.9500
N1—C7	1.288 (2)	C17—C18	1.428 (2)
N1—C8	1.467 (2)	C18—H18	0.9500
N1—H1N	0.885 (10)	C19—C21	1.529 (2)
N2—C18	1.297 (2)	C19—C22	1.532 (2)
N2—C19	1.468 (2)	C19—C20	1.539 (2)
N2—H2N	0.886 (10)	C20—H20A	0.9900
C1—C2	1.418 (2)	C20—H20B	0.9900
C1—C6	1.430 (2)	C20—H20C	0.9900
C2—C3	1.370 (3)	C20—H20D	0.9900
C2—H2A	0.9500	C21—H21A	0.9900
C3—C4	1.400 (3)	C21—H21B	0.9900
C3—H3A	0.9500	C21—H21C	0.9900
C4—C5	1.368 (2)	C21—H21D	0.9900
C5—C6	1.405 (2)	C22—H22A	0.9900
C5—H5	0.9500	C22—H22B	0.9900
			
C9—O2—H2	109.5	H10E—C10—H10F	105.1
C9—O2'—H2'	109.5	O4'—C11—O4	103.6 (2)
C9—O2"—H2"	109.5	O4'—C11—C8	123.9 (2)
C10—O3—H3	109.5	O4—C11—C8	107.90 (16)
C10—O3'—H3'	109.5	O4—C11—H11A	110.1
C10—O3"—H3"	109.5	C8—C11—H11A	110.1
C11—O4—H4	109.5	O4'—C11—H11B	100.6
C11—O4'—H4'	109.5	O4—C11—H11B	110.1
C20—O6—H6	109.5	C8—C11—H11B	110.1
C20—O6'—H6'	109.5	H11A—C11—H11B	108.4
C21—O7—H7	109.5	O4'—C11—H11C	106.4
C21—O7'—H7'	109.5	O4—C11—H11C	108.0
C22—O8—H8	109.5	C8—C11—H11C	106.4
C7—N1—C8	127.86 (14)	O4'—C11—H11D	106.4
C7—N1—H1N	112.8 (18)	C8—C11—H11D	106.4
C8—N1—H1N	119.1 (18)	H11B—C11—H11D	108.8
C18—N2—C19	127.48 (14)	H11C—C11—H11D	106.4
C18—N2—H2N	114.0 (16)	O5—C12—C13	122.12 (16)
C19—N2—H2N	118.0 (16)	O5—C12—C17	121.79 (15)
O1—C1—C2	121.63 (15)	C13—C12—C17	116.09 (17)
O1—C1—C6	121.54 (15)	C14—C13—C12	121.68 (18)
C2—C1—C6	116.83 (16)	C14—C13—H13	119.2
C3—C2—C1	121.40 (16)	C12—C13—H13	119.2
C3—C2—H2A	119.3	C13—C14—C15	120.98 (17)
C1—C2—H2A	119.3	C13—C14—H14	119.5
C2—C3—C4	120.73 (17)	C15—C14—H14	119.5
C2—C3—H3A	119.6	C16—C15—C14	120.36 (19)
C4—C3—H3A	119.6	C16—C15—Cl2	120.69 (16)
C5—C4—C3	120.11 (17)	C14—C15—Cl2	118.94 (14)
C5—C4—Cl1	120.71 (14)	C15—C16—C17	119.69 (17)
C3—C4—Cl1	119.18 (14)	C15—C16—H16	120.2
C4—C5—C6	120.31 (16)	C17—C16—H16	120.2
C4—C5—H5	119.8	C16—C17—C18	118.51 (15)
C6—C5—H5	119.8	C16—C17—C12	121.20 (15)
C5—C6—C1	120.61 (15)	C18—C17—C12	120.24 (16)
C5—C6—C7	118.89 (15)	N2—C18—C17	121.93 (15)
C1—C6—C7	120.46 (15)	N2—C18—H18	119.0
N1—C7—C6	122.31 (15)	C17—C18—H18	119.0
N1—C7—H7A	118.8	N2—C19—C21	111.20 (13)
C6—C7—H7A	118.8	N2—C19—C22	106.69 (13)
N1—C8—C11	112.26 (14)	C21—C19—C22	109.93 (14)
N1—C8—C10	105.88 (15)	N2—C19—C20	107.43 (13)
C11—C8—C10	109.77 (15)	C21—C19—C20	111.18 (14)
N1—C8—C9	108.14 (14)	C22—C19—C20	110.29 (14)
C11—C8—C9	110.66 (16)	O6'—C20—O6	105.2 (4)
C10—C8—C9	110.00 (16)	O6'—C20—C19	114.2 (3)
O2'—C9—C8	124.6 (2)	O6—C20—C19	111.78 (14)
O2"—C9—C8	104.9 (4)	O6—C20—H20A	109.3
O2—C9—C8	119.54 (19)	C19—C20—H20A	109.3
O2'—C9—H9A	103.4	O6'—C20—H20B	106.9
O2—C9—H9A	107.4	O6—C20—H20B	109.3
C8—C9—H9A	107.4	C19—C20—H20B	109.3
O2"—C9—H9B	118.1	H20A—C20—H20B	107.9
O2—C9—H9B	107.4	O6'—C20—H20C	108.7
C8—C9—H9B	107.4	O6—C20—H20C	108.0
H9A—C9—H9B	107.0	C19—C20—H20C	108.7
O2'—C9—H9C	106.2	H20A—C20—H20C	109.8
C8—C9—H9C	106.2	O6'—C20—H20D	108.7
H9A—C9—H9C	108.2	C19—C20—H20D	108.7
H9B—C9—H9C	120.1	H20A—C20—H20D	112.6
O2'—C9—H9D	106.2	H20B—C20—H20D	109.0
O2—C9—H9D	106.2	H20C—C20—H20D	107.6
C8—C9—H9D	106.2	O7—C21—O7'	101.9 (3)
H9B—C9—H9D	109.8	O7—C21—C19	108.48 (14)
O2"—C9—H9E	110.8	O7'—C21—C19	115.5 (3)
O2—C9—H9E	110.1	O7—C21—H21A	110.0
C8—C9—H9E	110.8	C19—C21—H21A	110.0
O2"—C9—H9F	110.8	O7—C21—H21B	110.0
C8—C9—H9F	110.8	O7'—C21—H21B	110.7
H9E—C9—H9F	108.8	C19—C21—H21B	110.0
O3'—C10—C8	116.5 (5)	H21A—C21—H21B	108.4
O3—C10—C8	111.74 (16)	O7—C21—H21C	114.3
O3—C10—H10A	109.3	O7'—C21—H21C	108.4
C8—C10—H10A	109.3	C19—C21—H21C	108.4
O3—C10—H10B	109.3	H21A—C21—H21C	105.6
C8—C10—H10B	109.3	O7'—C21—H21D	108.4
H10A—C10—H10B	107.9	C19—C21—H21D	108.4
O3'—C10—H10C	108.2	H21A—C21—H21D	116.7
C8—C10—H10C	108.2	H21B—C21—H21D	103.1
O3'—C10—H10D	108.2	H21C—C21—H21D	107.5
C8—C10—H10D	108.2	O8—C22—C19	109.62 (14)
H10C—C10—H10D	107.3	O8—C22—H22A	109.7
C8—C10—H10E	103.1	C19—C22—H22A	109.7
O3"—C10—H10F	103.1	O8—C22—H22B	109.7
O3'—C10—H10F	104.6	C19—C22—H22B	109.7
O3—C10—H10F	101.4	H22A—C22—H22B	108.2
C8—C10—H10F	103.1		
			
O1—C1—C2—C3	179.32 (17)	C9—C8—C11—O4'	58.1 (3)
C6—C1—C2—C3	−0.5 (3)	N1—C8—C11—O4	176.23 (16)
C1—C2—C3—C4	0.0 (3)	C10—C8—C11—O4	58.8 (2)
C2—C3—C4—C5	0.8 (3)	C9—C8—C11—O4	−62.8 (2)
C2—C3—C4—Cl1	−178.35 (15)	O5—C12—C13—C14	−179.70 (17)
C3—C4—C5—C6	−1.1 (3)	C17—C12—C13—C14	0.0 (3)
Cl1—C4—C5—C6	178.02 (13)	C12—C13—C14—C15	−0.3 (3)
C4—C5—C6—C1	0.6 (3)	C13—C14—C15—C16	−0.2 (3)
C4—C5—C6—C7	−177.26 (16)	C13—C14—C15—Cl2	178.80 (15)
O1—C1—C6—C5	−179.64 (15)	C14—C15—C16—C17	1.0 (3)
C2—C1—C6—C5	0.2 (2)	Cl2—C15—C16—C17	−177.95 (13)
O1—C1—C6—C7	−1.8 (2)	C15—C16—C17—C18	176.05 (16)
C2—C1—C6—C7	178.03 (16)	C15—C16—C17—C12	−1.4 (3)
C8—N1—C7—C6	−174.53 (16)	O5—C12—C17—C16	−179.45 (15)
C5—C6—C7—N1	178.05 (16)	C13—C12—C17—C16	0.9 (2)
C1—C6—C7—N1	0.2 (3)	O5—C12—C17—C18	3.2 (2)
C7—N1—C8—C11	7.8 (2)	C13—C12—C17—C18	−176.51 (15)
C7—N1—C8—C10	127.59 (19)	C19—N2—C18—C17	171.78 (15)
C7—N1—C8—C9	−114.5 (2)	C16—C17—C18—N2	−179.76 (15)
N1—C8—C9—O2'	−52.8 (3)	C12—C17—C18—N2	−2.3 (2)
C11—C8—C9—O2'	−176.2 (3)	C18—N2—C19—C21	36.7 (2)
C10—C8—C9—O2'	62.4 (3)	C18—N2—C19—C22	156.57 (16)
N1—C8—C9—O2"	174.6 (5)	C18—N2—C19—C20	−85.16 (19)
C11—C8—C9—O2"	51.2 (5)	N2—C19—C20—O6'	−177.7 (4)
C10—C8—C9—O2"	−70.2 (5)	C21—C19—C20—O6'	60.4 (4)
N1—C8—C9—O2	63.7 (2)	C22—C19—C20—O6'	−61.8 (4)
C11—C8—C9—O2	−59.6 (2)	N2—C19—C20—O6	62.94 (17)
C10—C8—C9—O2	178.90 (19)	C21—C19—C20—O6	−58.93 (18)
N1—C8—C10—O3"	−80.6 (7)	C22—C19—C20—O6	178.86 (14)
C11—C8—C10—O3"	40.8 (7)	N2—C19—C21—O7	175.00 (14)
C9—C8—C10—O3"	162.8 (7)	C22—C19—C21—O7	57.08 (18)
N1—C8—C10—O3'	−92.1 (5)	C20—C19—C21—O7	−65.33 (18)
C11—C8—C10—O3'	29.3 (5)	N2—C19—C21—O7'	−71.5 (4)
C9—C8—C10—O3'	151.3 (4)	C22—C19—C21—O7'	170.6 (4)
N1—C8—C10—O3	46.0 (2)	C20—C19—C21—O7'	48.2 (4)
C11—C8—C10—O3	167.35 (18)	N2—C19—C22—O8	48.05 (18)
C9—C8—C10—O3	−70.7 (2)	C21—C19—C22—O8	168.73 (14)
N1—C8—C11—O4'	−62.9 (3)	C20—C19—C22—O8	−68.33 (18)
C10—C8—C11—O4'	179.7 (3)		

### Hydrogen-bond geometry (Å, °)

**Table:**

*D*—H···*A*	*D*—H	H···*A*	*D*···*A*	*D*—H···*A*
N1—H1n···O1	0.87 (1)	1.82 (2)	2.582 (2)	143 (2)
N2—H2n···O5	0.87 (1)	1.82 (2)	2.573 (2)	141 (2)
